# Graphene-Iron Ore Tailings–Based Cementitious Composites with High Early Flexural Strength

**DOI:** 10.3390/ma16010327

**Published:** 2022-12-29

**Authors:** Huiteng Xiao, Na Zhang, Gen Li, Youpeng Zhang, Yidi Wang, Yu Wang, Yihe Zhang

**Affiliations:** 1 Engineering Research Center of Ministry of Education for Geological Carbon Storage and Low Carbon Utilization of Resources, China University of Geosciences (Beijing), Beijing 100083, China; 2 Beijing Key Laboratory of Materials Utilization of Nonmetallic Minerals and Solid Wastes, China University of Geosciences (Beijing), Beijing 100083, China; 3 National Laboratory of Mineral Materials, China University of Geosciences (Beijing), Beijing 100083, China; 4 School of Materials Science and Technology, China University of Geosciences (Beijing), Beijing 100083, China

**Keywords:** iron ore tailings, cementitious composites, nitrogen-doped graphene, flexural strength, enhancement mechanism

## Abstract

Graphene is a two-dimensional nanomaterial with excellent mechanical, electrical and thermal properties. The application of graphene in cement-based materials has good prospects. However, the mechanical properties of cement-based materials are difficult to be significantly enhanced by ordinary graphene nanoplates. In this paper, nitrogen-doped graphene is first reported as an additive with dosages of 0.01, 0.02, 0.03, 0.04 and 0.05 wt.%, respectively, to prepare iron ore tailings–based cementitious composites. The iron ore tailings–based cementitious composite with 0.02 wt.% graphene shows an extremely high flexural strength of 15.05 MPa at 3 days, which is 134.4% higher than that of the iron ore tailings–based cementitious composite without graphene. The effects of graphene content and curing age on the flexural strength and microstructure of iron ore tailings–based cementitious composites were studied. In particular, the scanning electron microscope was adopted to observe the micromorphology of the composites. It is helpful to understand the graphene reinforcement mechanism for the high early flexural strength of iron ore tailings–based cementitious composites. By altering the morphology of iron ore tailings–based cementitious composites, graphene plays two roles in the composites. One role is to connect C-(A)-S-H gels, ettringite and other hydrated crystals to construct a three-dimensional structure. The other is to attract iron ore tailings distributed on its platform to enhance its flexural strength properties. These findings provide favorable guidance for the performance enhancement and mechanism replenishment of graphene-reinforced cementitious composites.

## 1. Introduction

Ordinary cement-based mortar and concrete materials are generally hard and brittle with low bending strength. It is difficult to meet the growing engineering needs [1,2]. To solve this problem, micro- and nanomaterials such as polypropylene (PP) fibers, basalt fibers, nano-silica, carbon nanotubes and graphene are often used as reinforcing materials to control the growth of cracks and enhance strength properties in cement-based composites materials [3,4,5,6,7,8]. Graphene is a new research target among these nanomaterials because of its excellent properties. As a typical two-dimensional nanomaterial, graphene has a hexagonal honeycomb lattice structure, which can be viewed as a layer of stripped graphite molecule. Each carbon atom adopts sp^2^ hybridization and contributes electrons to the remaining p orbital to form a π bond. π electrons can move freely. It endows graphene with good conductivity [9,10]. The connection between carbon atoms inside graphene is very flexible. When an external force is applied to graphene, the surface of carbon atoms will bend and deform. As the carbon atoms do not have to be rearranged under the external force, graphene maintains structural stability. Therefore, graphene has excellent tensile strength and elastic modulus [11].

Studies have shown that graphene and its derivatives have a certain attraction to cement hydration products, and they could be used to effectively strengthen cement-based composites [12,13,14]. Graphene oxide (GO) contains a lot of oxygen-containing functional groups [15]. Although the functional groups reduce part of the physical properties of GO, but they make GO have good dispersion and oxidation activity. Extensive research has demonstrated that GO is well dispersible in solution, and it has a great potential to interact with cement matrix [16,17,18,19,20,21,22]. In addition, reduced graphene oxide (rGO) not only has excellent properties of graphene but also contains some oxygen-containing functional groups. Moreover, rGO has a certain application in cement-based composites [23,24,25,26,27]. However, for graphene nanoplates, the low dispersibility in solution restricts their general application in cement-based composites. There is a common belief that graphene nanoplates (GNPs) can agglomerate and accumulate easily in cement matrix because of their high surface area and surface energy, resulting in an unremarkable reinforcement effect on cement-based composites [28,29,30,31]. Generally, GO is thought to be more appropriate for reinforcement in cement-based composites than GNPs are.

Table 1 shows the effects of graphene and its derivatives on the mechanical properties of cement-based composites in different studies [28,31,32,33,34,35,36,37,38,39,40]. In contrast, the reinforcement effect of GNPs on the mechanical properties of cementitious composites is not obvious. Du et al. [40] used naphthalene sulfonate–based surfactant to disperse the graphene nanoplates with a thickness of 37 nm. They found that the mechanical properties of concrete mixed with graphene nanoplates exhibited almost no change. Bai et al. [41] used graphene with a thickness of 1.0–1.77 nm to prepare cement mortar. They found that the compressive strength of mortar mixed with 2.0 wt.% graphene at 3 days and 28 days even decreased by 31% and 20%, respectively.

However, some recent studies have reported that graphene can well connect C-S-H gels, ettringite and other hydration products together to form a three-dimensional structure and can improve the mechanical properties of composites by filling pores and bridging cracks in the cementitious system [34,42]. Silva et al. [43] found that the splitting tensile strength and compressive strength increased by 131.6% and 95.7%, respectively, at 28 days in the composites prepared by adding multilayer graphene. Tong et al. [31] found that mortar mixed with 0.1 wt.% GNPs showed a better compressive strength enhancement effect, which increased by 19.9% compared with the control group. Han et al. [44] found that the addition of multilayer graphene could increase the compressive strength and flexural strength of cement-based composites by 54% and 21%, respectively. Cao et al. [45] mixed 0.02 wt.% functionalized graphene nanoplates into cement mortar. They found that the 28-day flexural strength increased by 32.0% and that compressive strength increased by 20.3%. Apparently, to optimize the benefits and general utilization of graphene in construction and building materials, further research is still necessary to investigate the enhancement mechanism of graphene in different cementitious composites.

In addition, the production of cement and concrete industries faces problems such as resource depletion and high energy consumption. In response to this problem, many researchers are committed to studying how to effectively use solid wastes to prepare cement or concrete [46,47,48,49,50]. Iron ore tailings are solid wastes discharged from the iron ore beneficiation process [51]. In China, owing to the significant increase of steel production, huge amounts of iron ore tailings are produced every year. They are stockpiled as waste in tailings dams and ponds. Obviously, iron ore tailings not only occupy large areas of stockpiling sites but also cause serious environmental problems. In order to pursue the development concept of “lucid waters and lush mountains are invaluable assets”, it is urgent and necessary to largely utilize iron ore tailings to produce green materials. Iron ore tailings can be used as a fine aggregate to substitute river sand for the production of green and sustainable concrete [52,53,54,55]. Iron ore tailings are basically waste ground rocks with fine particle size. They are relatively inert with a main mineral phase of quartz [56]. Zhao et al. [57] prepared ultrahigh-performance concrete (UHPC) with P.II 52.5 ordinary Portland cement and silicone fume as raw materials and with natural river sand (RS) and iron ore tailing sand (TS) as aggregates. It was found that the compressive strength of UHPC still reached 120 MPa when the substitution rate of iron ore tailings for river sand reached 50%. Tian et al. [52] prepared concrete by replacing 35% of natural sand aggregate with iron ore tailings. Its mechanical properties were basically equivalent to the concrete made of natural sand. It is of great significance to apply iron ore tailings as a fine aggregate in mortar and concrete materials. This not only saves the area of stockpiling sites and alleviates the environmental burdens of iron ore tailings but also largely decreases the consumption of river sand, and it further solves the destruction problem of river embankment [58].

In this paper, nitrogen-doped graphene was used as an additive with dosages of 0.01, 0.02, 0.03, 0.04 and 0.05 wt.%, respectively, to reinforce iron ore tailings–based cementitious composites, which were prepared by a cement and iron ore tailings aggregate. Their mechanical properties were evaluated by conducting flexural and compressive strengths tests. It was found that compared with the standard sand-cement cementitious system, the early flexural strength of iron ore tailings–based cementitious composites was improved more significantly with the addition of graphene. The microstructure of graphene and its reinforced iron ore tailings–based cementitious composites were observed by a scanning electron microscope (SEM). A new reinforcement mechanism is proposed to support the high early flexural strength of graphene-iron ore tailings–based cementitious composites.

## 2. Experimental Section

### 2.1. Raw Materials

The raw materials for producing graphene-iron ore tailings–based cementitious composites (GICC) are nitrogen-doped graphene, iron ore tailings, Portland cement and Belite cement. In addition, polycarboxylate superplasticizer was adopted as a strengthening agent. It also played an important role in dispersing the graphene [8].

Graphene was provided by Guangdong Minglu Tianyuan New Material Technology Co., Ltd. (Foshan, China). It consists of 1~5 layers. Its packing density is 0.08~0.12 g·cm^−3^, and the particle size is 6~60 μm. The chemical composition is mainly C and O.

**Figure 1:**
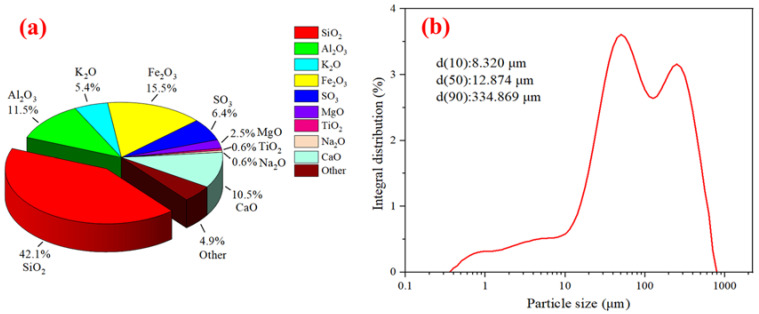
Chemical composition and particle-size distribution of iron ore tailings: (**a**) chemical composition and (**b**) particle-size distribution.

**Figure 2:**
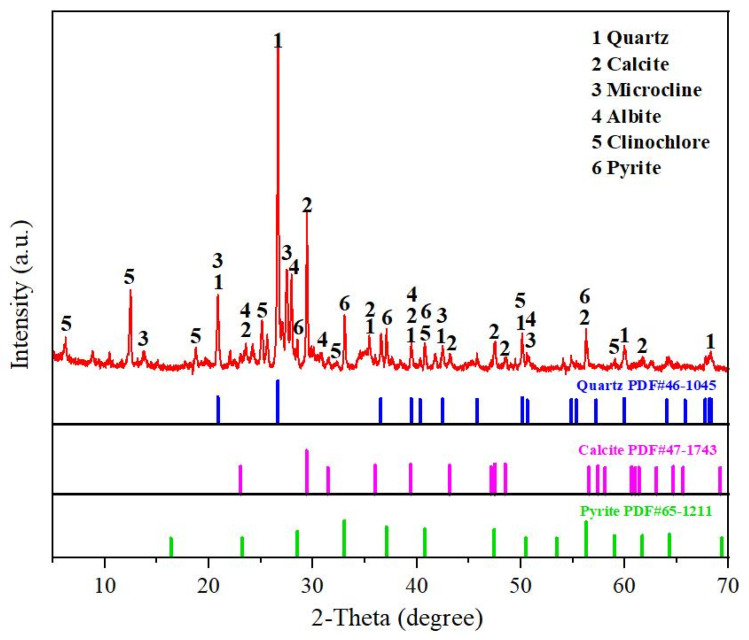
XRD pattern of iron ore tailings.

Iron ore tailings were obtained from Ili Kazak Autonomous Prefecture of Xinjiang, China. Figure 1 shows the chemical composition and the particle-size distribution of iron ore tailings. The particle size of iron ore tailings is distributed mainly in the following range: 0.356~709.627 μm. Its chemical composition includes mainly SiO_2_, Fe_2_O_3_, Al_2_O_3_ and CaO. Figure 2 shows the XRD pattern of iron ore tailings. Its main mineral components are quartz, calcite, microcline, albite, clinochlore and pyrite.

Portland cement was supplied by Tangshan Jidong Cement Co., Ltd. (Tangshan, China). Its Blaine specific surface area and specific gravity are 390 m^2^/kg and 3.15 g/cm^3^, respectively. Belite cement was supplied by Tangshan Polar Bear Building Materials Co., Ltd. (Tangshan, China). Its Blaine specific surface area and specific gravity are 450 m^2^/kg and 2.97 g/cm^3^, respectively. Table 2 lists the main chemical components of Portland cement and Belite cement.

Polycarboxylate superplasticizer was supplied by Qinfen Building Materials Co., Ltd. (Weinan, China). It is in a liquid form with a water reduction rate of 28%, and its gas content is 5%.

### 2.2. Preparation of Graphene-Iron Ore Tailings–Based Cementitious Composites

**Figure 3:**
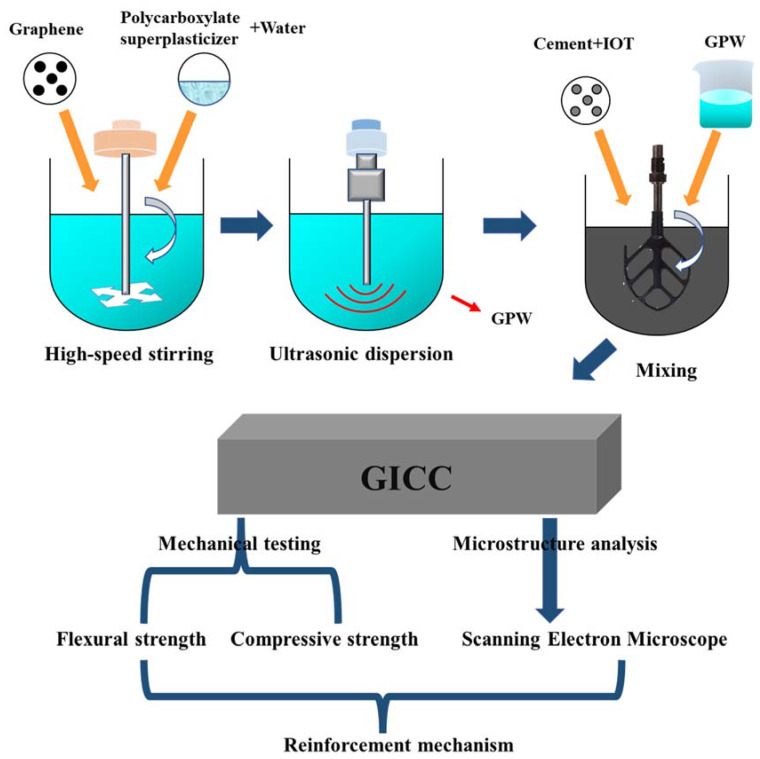
Overall research flow of graphene-iron ore tailings–based cementitious composites.

The experimental materials were weighed at the proportions shown in Table 3. Figure 3 presents the preparation process of graphene-iron ore tailings–based cementitious composites (GICC). First, under the condition that the dosage of polycarboxylate superplasticizer agent was 0.1 wt.% of the cement mass, we prepared cement-iron ore tailings mortars with a flowability of 190~195 mm to obtain the best water–cement ratio of 0.28. According to our previous studies [42,59], the weight ratio of Belite cement to Portland cement was 0.25, if the aim is to greatly reduce the shrinkage and cracking of the hardened cementitious composites. Then, polycarboxylate superplasticizer, graphene and water were mixed and stirred in a beaker for 5 min. The parameters of the ultrasonic dispersion instrument (FS-600, Shanghai Shengxi Ultrasonic Instrument Co., Ltd., Shanghai, China) were set at 80% power and 45 °C, and the ultrasonic dispersion was conducted for 30 min to obtain a fully dispersed graphene mixed solution (GPW). Finally, GPW solution, cement and iron ore tailings were mixed and stirred in a mortar mixer to prepare the cementitious composites.

The GICC mixture was stirred by the mortar mixer slowly at 75 rpm for 30 s, and then stirred at a fast speed of 300 rpm for 90 s. Subsequently, the mixture was poured into a standard mold of 40 mm × 40 mm × 160 mm. The mixture in the mold was vibrated for 120 s with a shaking table to expel the entrapped air from the mortar. Finally, plastic wrap was used to prevent moisture from evaporating from the mortar. The samples were demolded after 24 h and cured at 20 ± 2 °C with a relative humidity of 95%. To obtain representative mechanical strength measurements, five replicates were performed for each batch.

### 2.3. Testing Methods

#### 2.3.1. Phase Analysis of Iron Ore Tailings

The mineralogical components of iron ore tailings were analyzed by X-ray powder diffraction (XRD) (Bruker D8 Advance Instrument, Karlsruhe, Germany) using Cu Kα Radiation (λ = 1.54056 Å) at 40 kV and 40 mA. The scanning range was 5°~70°, and the scanning speed was 6°/min.

#### 2.3.2. Compressive and Flexural Strengths Test

The mechanical properties test was carried out according to GB/T 17671-1999 [60]. The flexural strength of the GICC mortar samples was tested by using a three-point bending tester at a loading rate of 50 N/s. A hydraulic universal testing machine (300 kN) was adopted to test the compressive strength, in which the loading area was 40 mm × 40 mm and the loading rate was 2.4 kN/s.

#### 2.3.3. Microstructure Test

After the mechanical properties test, the typical samples were analyzed by a SU 8020 field-emission-scanning electron microscope (Hitachi, Tokyo, Japan). Before the SEM characterization, the selected samples were soaked with ethanol for 72 h to stop the hydration process, and then the samples were placed in an oven at 55 °C, drying for 12 h to eliminate the ethanol. Before SEM images were taken, the samples were treated with gold sputtering to improve the SEM image quality.

## 3. Results and Discussion

### 3.1. Flexural and Compressive Strengths

**Figure 4:**
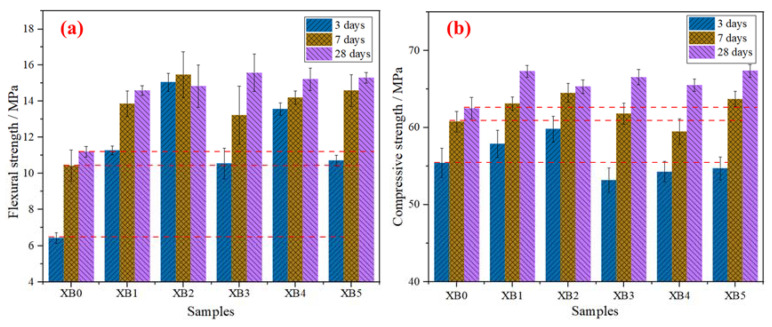
Effect of graphene content on mechanical properties of GICC: (**a**) flexural strength and (**b**) compressive strength.

In order to study the reinforcing effect of graphene on iron ore tailings–based cementitious composites, the mechanical properties of composites before and after adding graphene were analyzed. The effect of graphene content on the flexural and compressive strengths of GICC is presented in Figure 4.

As shown in Figure 4a, it is obvious that the flexural strength of the XB1~XB5 samples composed of graphene is greatly improved compared with the XB0 sample without graphene. When the graphene dosage is 0.02 wt.%, the improvement of GICC is the most significant at the early age. The 3-day flexural strength of the XB2 sample increased by 134.4%. Its 7-day flexural strength and its 28-day flexural strength increased by 48.3% and 32.5%, respectively. The standard deviations of the samples all meet the requirements of the GB/T 17671-1999 standard. The highest standard deviation does not exceed 1.9, which proves that the gap between the different samples is small and the data are reliable.

As shown in Figure 4b, the compressive strength basically increases with the curing time. However, the compressive strengths of the GICC samples change little after adding graphene. The standard deviation of each group of data meets the national standard of GB/T 17671-1999. The standard deviation is also small, indicating that the compressive strength of the material is uniform and reliable.

Table 4 lists the mechanical properties of iron ore tailings–based cementitious materials without graphene that were previously studied by our research group. Compared with these results, our present work still shows great advancement in flexural strength. The 3-day flexural strength of iron ore tailings–based cementitious composites without graphene exceeds 6 MPa, and the 7-day flexural strength exceeds 10 MPa. After adding graphene in the iron ore tailings–based cementitious composites, the flexural strength is more significantly enhanced. The lowest 3-day flexural strength of the GICC sample can attain 10 MPa.

**Figure 5:**
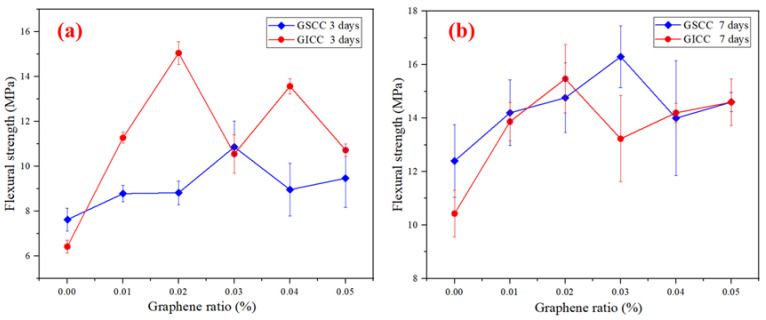
Flexural strength of GSCC and GICC: (**a**) 3 days and (**b**) 7 days.

Figure 5 shows a comparison of the flexural strengths between graphene-standard sand-cement composites (GSCC) and graphene-iron ore tailings–based cementitious composites (GICC). The microstructure and mechanical properties of GSCC were investigated in our previous work, which was reported in [42]. It is noticed that without graphene, the flexural strength of GICC is lower than that of GSCC. Compared with GSCC, the 3-day and 7-day flexural strengths of GICC are significantly improved when adding 0.02 wt.% graphene. Therefore, a graphene dosage of 0.02 wt.% is obviously more suitable to enhance the flexural strength of GICC.

### 3.2. Microstructure Analysis

#### 3.2.1. The Effect of Raw Materials

**Figure 6:**
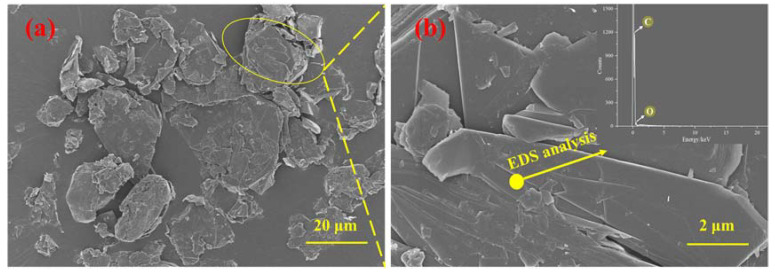
SEM-EDS analysis of nitrogen-doped graphene nanoplates: (**a**) SEM image and (**b**) EDS result.

Figure 6 and Table 5 show the SEM images and EDS results of the nitrogen-doped graphene nanoplates used in the experiment. The C/O ratio of graphene is 91:9, which is close to that of the reported nitrogen-doped graphene [63,64]. The graphene sheets have a width of 10~15 μm, a length of 20~30 μm and a thickness of 15~20 nm. They are easy to aggregate, and the surface contains a wrinkled texture. Using polycarboxylate superplasticizer solution can effectively disperse the graphene nanoplates [42].

**Figure 7:**
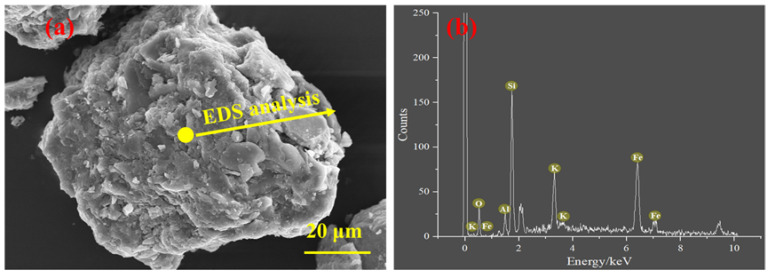
SEM-EDS analysis of iron ore tailings: (**a**) SEM image and (**b**) EDS result.

Given the complex composition of iron ore tailings, an SEM-EDS test was carried out for iron ore tailings. The micromorphology and element composition of iron ore tailings are presented in Figure 7. It can be seen that the various crystals that accumulated in iron ore tailings are relatively loose. Additionally, a large number of irregular fine particles are deposited on the surface of large crystals [65]. The EDS result of iron ore tailings basically corresponds to the main chemical composition presented in Figure 1a.

**Figure 8:**
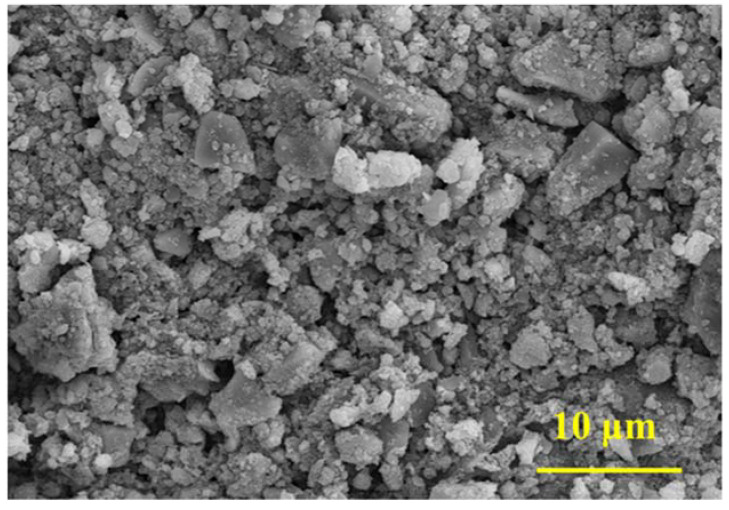
SEM image of iron ore tailings–based cementitious composite (XB0 sample).

The micromorphology of an iron ore tailings–based cementitious composite (the XB0 sample) is shown in Figure 8. A large number of irregular fine particles are deposited onto the surface of the cement matrix, which is similar to the micromorphology of iron ore tailings. Crystals with different sizes are loosely deposited onto the surface of the cement matrix, forming a large number of pores and destroying the compact matrix. This is one of the reasons why the flexural strength of iron ore tailings–based cementitious composite is lower than that of standard sand-cement composites [40]. Under the action of an external force, a large number of pores are generated inside the cement matrix thanks to the loose accumulation of iron ore tailings. Moreover, microcracks are easily generated and expanded, eventually leading to the fracturing of the composite [66,67].

**Figure 9:**
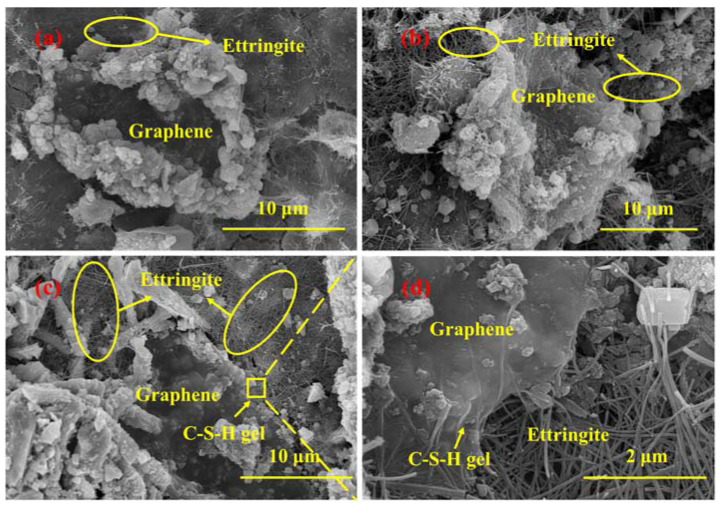
SEM images of XB2 (graphene dosage of 0.02 wt.%): (**a**) hydrated for 3 days; (**b**) hydrated for 7 days; (**c**) hydrated for 28 days; (**d**) hydrated for 28 days.

As shown in Figure 4a, the flexural strength of GICC is greatly improved in a short time after adding graphene into the iron ore tailings–based cementitious composites. The graphene distribution and its cross-linking form with the matrix can be observed in the SEM images shown in Figure 9. A three-dimensional structure similar to the GSCC system can be observed in the micromorphology of the XB2 sample with a graphene dosage of 0.02 wt.% [42].

However, the graphene-iron ore tailings–based cementitious composite is different from the graphene-standard sand-cement composite. It can be seen in Figure 9a that lots of large particles are deposited onto the surface of graphene. These particles are not hydration products of cement. They are individual particles with different sizes and shapes contained in iron ore tailings. Although iron ore tailings are used as an aggregate in GICC, clay minerals such as clinochlore in the iron ore tailings could release active Si and Al to react with Ca(OH)_2_ to form C-(A)-S-H gels [68,69]. Some previous studies have also shown the formation of C-S-H gel and C-(A)-S-H gel [42,62,70]. The iron ore tailings particles deposit onto the edge of graphene thanks mainly to the binding of C-S-H gels. They are surrounded by a lot of ettringite.

#### 3.2.2. The Effect of Graphene Content

**Figure 10:**
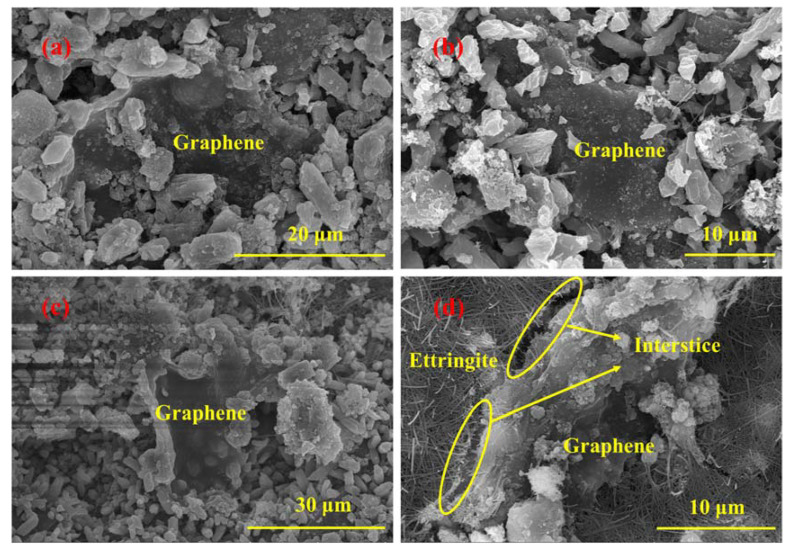
SEM images of GICC hydrated for 28 days with different graphene contents: (**a**) XB3 (graphene dosage of 0.03 wt.%); (**b**) XB4 (graphene dosage of 0.04 wt.%); (**c**) XB5 (graphene dosage of 0.05 wt.%); (**d**) XB5 (graphene dosage of 0.05 wt.%).

Figure 10 shows the SEM images of the XB3, XB4 and XB5 samples hydrated for 28 days. When the graphene dosage is 0.03 wt.%, 0.04 wt.% and 0.05 wt.%, a large number of irregularly shaped and sized bulk crystals deposit around the graphene, the morphology of which is similar to the iron ore tailings. It indicates that with the increasing content of graphene, more and more iron ore tailings tend to deposit onto the surface of graphene. The graphene edges could not contact the ettringite, thus losing the connection with the cement matrix [42]. On one hand, the graphene would be isolated in the cement matrix. On the other hand, some voids are generated in this process, and the compactness of the cement matrix decreases [51]. Graphene can bridge and fill the pores in the cement matrix, but it cannot form an effective three-dimensional structure to continually improve the flexural strength of GICC when the graphene addition is excessive. As an appropriate amount of iron ore tailings deposit onto the graphene, free active Si and Al further generate C-(A)-S-H gels. The three-dimensional structure of graphene-ettringite-C-S-H gel can be effectively formed in the composite of the XB2 sample. Hence, the flexural strength of the XB2 sample is rapidly improved in a short hydration time. In other samples, however, the final change in flexural strength is smaller owing to the weakness of the three-dimensional structure resulting from a large number of iron ore tailings depositing onto the graphene. It is thought that the formation of an effective three-dimensional structure of graphene-ettringite-C-S-H gel is an important reason for improving the flexural strength of GICC.

#### 3.2.3. The Effect of Hydration Age

**Figure 11:**
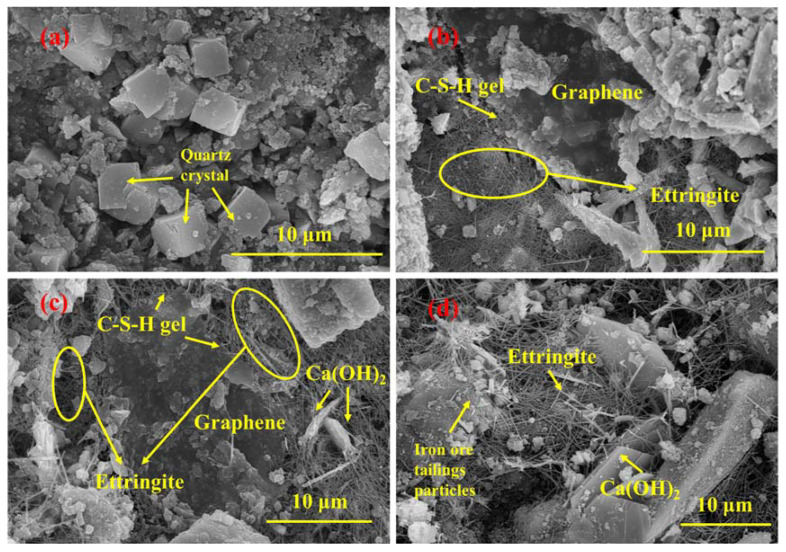
SEM images of XB2: (**a**) hydrated for 3 days; (**b**) hydrated for 7 days; (**c**) hydrated for 28 days; (**d**) hydrated for 28 days.

Figure 11 shows the SEM images of the XB2 sample with a different hydration age. With the prolongation of hydration time, it seems that the amount of ettringite around graphene significantly increases. Ettringite contributes to the early strength of cement [71]. At the same time, other hydration products also gradually appear. Silica crystals can be observed in Figure 11a, and hexagonal crystals of Ca(OH)_2_ are also found in the 28-day hydrated sample in Figure 11d. Meanwhile, ettringite covered by C-S-H gel on graphene sheets can also be observed in Figure 11c.

As shown in Figure 11c, Ca(OH)_2_ is also linked to graphene through the bridging effect of ettringite. A tight connection system established by the ettringite meshes connects various hydrated crystals with graphene sheets to enhance the strength of cementitious composites. It can also be seen that the C-S-H gels increase with the prolongation of hydration time. In the interface transition zone between the graphene and the ettringite, some deposited crystals and ettringite are covered by C-(A)-S-H gels, further consolidating the composite structure [70]. With the increase of hydration time, the three-dimensional structure of graphene-ettringite-C-(A)-S-H gel is constantly enriched and improved, thereby further enhancing the flexural strength of GICC.

### 3.3. Flexural Strength Reinforcement Mechanism

It can be seen from the XB2 sample that the three-dimensional structure theory found in the previous GSCC is also applicable to GICC [42]. Both of them are composed of graphene-ettringite-C-S-H gel to form a regular three-dimensional system. However, what makes GICC different from the three-dimensional structure of GSCC is that iron ore tailings are likely to deposit onto the graphene surface [42,72]. When the graphene dosage does not exceed 0.02 wt.%, the appropriate amount of iron tailings deposited onto the graphene can solidify the three-dimensional structure.

**Figure 12:**
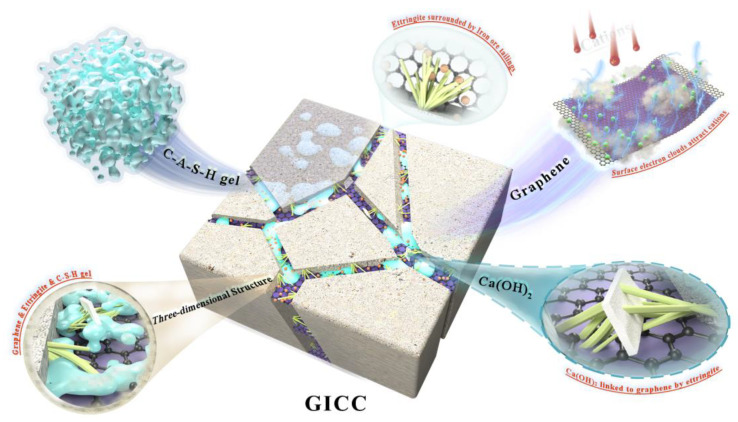
Flexural strength reinforcement mechanism of GICC.

The flexural strength enhancement mechanism of GICC is presented in Figure 12. First, graphene fills the pores or cracks in the cement matrix to act as a bridge. Second, ettringite forms a bridge between the cement matrix and the graphene, firmly connecting the graphene and the cement matrix. This can transfer the applied load to the graphene surface, so that graphene can further bear the applied load. At the same time, ettringite also connects Ca(OH)_2_ with graphene to enhance the flexural strength of GICC. The main hydration product, C-(A)-S-H gels, not only can make the connection between the graphene, iron ore tailings and the ettringite tighter but also can play a fixed role that makes the connection between the graphene and the substrate stronger. In addition, adding an appropriate amount of graphene can effectively deposit iron ore tailings particles onto its surface, because they show a tendency to attract each other [73]. The fine and low-activity particles of iron ore tailings are bonded to the C-(A)-S-H gels and the ettringite in the cement critical zone, which can further enhance the early flexural strength of GICC.

Finally, graphene nanoplates have large specific surface areas and flat planes. Their dense electron clouds lead to the absorption of cations, thus providing a substrate for crystal formation and growth [42]. A moderate amount of iron ore tailings deposit onto the surface of the graphene, solidifying the three-dimensional structure. The excellent mechanical properties of graphene are used to protect the cement matrix, thereby improving the mechanical properties of GICC.

## 4. Conclusions

In this paper, the flexural strength development and microstructure of iron ore tailings–based cementitious composites (GICC) were deeply investigated. Additionally, the flexural strength reinforcement mechanism of GICC was discussed and clarified. The following conclusions were drawn:

(1) The early mechanical properties of iron ore tailings–based cementitious composites were enhanced by using graphene. Graphene showed a great effect on the flexural strength development of iron ore tailings–based cementitious composites while keeping the compressive strength basically unchanged.
(2) Graphene can significantly improve the flexural strength of iron ore tailings–based cementitious composites in a short hydration time. When adding a 0.02 wt.% dosage of graphene, the 3-day flexural strength of GICC attained 15.05 MPa. It was 134.4% higher than that of the iron ore tailings–based cementitious composite without graphene.
(3) There was a three-dimensional structure of graphene-ettringite-C-(A)-S-H gel in the GICC system. This structure can effectively improve the flexural strength of iron ore tailings–based cementitious composites.
(4) A proper amount of iron ore tailings deposited onto the graphene can solidify the three-dimensional structure. However, when the graphene content exceeded 0.02 wt.%, large amounts of iron ore tailings were likely to deposit onto the graphene surface. They separated the graphene from the cement matrix, thus reducing the flexural strength of GICC.

Our results showed that nitrogen-doped graphene had a greater effect on improving the early flexural strength of iron ore tailings–based cementitious composites. At present, the research and application of graphene nanoplates in solid wastes-based cementitious composites are still few. The development of long-term mechanical properties and the quantitative analysis of the cementitious components in the nitrogen-doped graphene-reinforced cementitious composites should be further investigated in our future work.

## Figures and Tables

**Table 1:** Effects of graphene and its derivatives on the mechanical properties of cementitious composites in different studies.

Matrix	Type/Dosage (wt.%)	w/b	Increase in Flexural Strength (%)/Age	Increase in Compressive Strength (%)/Age	Ref.
Paste	GO/0.05	0.29	69.4/7 days	66.4/7 days	[28]
Paste	GO/0.022	0.42	26.7/3 days	27.6/3 days	[32]
Paste	rGO/0.02	0.32	70.0/7 days	22.0/28 days	[33]
Paste	GNPs/0.15	0.30	27.5/28 days	49.4/28 days	[34]
Paste	GNPs/0.06	0.35	22.2/7 days	16.7/7 days	[35]
Mortar	GO/0.08	0.20	80.6/8 days	–	[36]
Mortar	rGO/0.10	0.485	–	57.5/7 days	[37]
Mortar	GNPs/0.10	0.54	–	19.9/28 days	[31]
Concrete	GO/0.10	0.50	~13.0/7 days	~47.0/7 days	[38]
Concrete	rGO/0.05	0.43	36.0/28 days	113.0/3 days	[39]
Concrete	GNPs/2.50	0.47	–	Almost unchanged/28 days	[40]

Here, w/b is the water-binder ratio.

**Table 2:** Chemical composition of ordinary Portland cement and Belite cement (wt.%).

Materials	SiO_2_	CaO	MgO	Al_2_O_3_	SO_3_	Fe_2_O_3_
Ordinary Portland cement	24.85	48.38	4.39	10.88	2.95	2.68
Belite cement	16.85	48.71	2.40	14.88	11.95	1.47

**Table 3:** The mixing ratio of GICC mortars.

Sample	Iron Ore Tailings/(wt.%)	Portland Cement/(wt.%)	Belite Cement/(wt.%)	Polycarboxylate Superplasticizer/(wt.%)	Graphene/(wt.%)
XB0	50	40	10	0.1	0.00
XB1	50	40	10	0.1	0.01
XB2	50	40	10	0.1	0.02
XB3	50	40	10	0.1	0.03
XB4	50	40	10	0.1	0.04
XB5	50	40	10	0.1	0.05

The total mass of cement (Portland cement and Belite cement) and iron tailings was 1800 g. Polycarboxylate superplasticizer and graphene were used as additives, and the adding ratio of polycarboxylate superplasticizer and graphene was the weight percentage of cement.

**Table 4:** Mechanical properties of cementitious materials composed of iron ore tailings in different studies.

Matrix	L/S	Aggregate	Flexural Strength (MPa)/Age	Compressive Strength (MPa)/Age	Ref.
Mortar	0.18	Iron ore tailings	6.32/7 days	32.14/7 days	[61]
Mortar	0.20	Iron ore tailings/copper tailings	6.80/3 days	27.6/3 days	[62]

L/S is the liquid–solid ratio.

**Table 5:** EDS result of graphene used in the experiment.

Element	Atomic/%	Weight/%
C	91.02	88.38
O	8.98	11.62

## Data Availability

Data will be made available on request.
